# Latent class analysis identifies three biologically distinct phenotypes among patients undergoing endovascular thrombectomy: a derivation and temporal validation study

**DOI:** 10.3389/fneur.2026.1855033

**Published:** 2026-10-15

**Authors:** Li-Li Yang, Chao Chen, Li-Li Sun, Meng-Xia Li, Li Han

**Affiliations:** Department of Neurology, Taizhou Hospital of Zhejiang Province, Affiliated to Wenzhou Medical University, Linhai, Zhejiang, China

**Keywords:** blood glucose, D-dimer, endovascular thrombectomy, functional outcome, latent class analysis, patient phenotypes

## Abstract

**Background:**

Despite successful vascular recanalization, approximately half of patients undergoing endovascular thrombectomy (EVT) fail to achieve functional independence at 3 months. The biological determinants of this heterogeneity remain poorly characterized.

**Methods:**

Latent class analysis (LCA) was applied to 825 consecutive EVT-treated patients (development cohort, January 2021–December 2024) using 12 routine post-procedural clinical and laboratory indicators. Phenotype associations with favorable 3-month outcome (modified Rankin Scale 0–2) were assessed using multivariable logistic regression. Reproducibility was evaluated in an independent temporal validation cohort (*n* = 211).

**Results:**

LCA identified three phenotypes. The Low-burden phenotype (Class 1, 44.4%) had the lowest inflammatory and coagulatory burden and achieved favorable outcome in 70.8% of patients. The Metabolic phenotype (Class 2, 42.7%), characterized by hyperglycemia and elevated neutrophil-to-lymphocyte ratio, had a favorable outcome rate of 44.9% and symptomatic intracranial hemorrhage (sICH) rate of 7.1%. The hypercoagulable phenotype (Class 3, 13.0%), defined by markedly elevated D-dimer and atrial fibrillation, had a favorable outcome rate of 40.2% and sICH rate of 8.4%. After multivariable adjustment, both Class 2 (OR 0.49, 95% CI 0.35–0.69) and Class 3 (OR 0.41, 95% CI 0.25–0.67) were independently associated with unfavorable outcome (both *p* < 0.001). Class distribution and outcome associations were reproduced in the validation cohort.

**Conclusion:**

LCA identified three biologically distinct phenotypes among EVT-treated patients with divergent functional outcomes. Multicenter prospective validation is needed before these findings can inform clinical practice.

## Introduction

Acute ischemic stroke remains a leading global cause of mortality and long-term disability, with an estimated 12.2 million incident strokes annually and more than 100 million people living with its consequences (1). Endovascular thrombectomy (EVT) has transformed the management of large vessel occlusion stroke, with multiple randomized controlled trials demonstrating substantial improvements in functional independence compared with medical therapy alone (2, 3). Despite successful vascular recanalization in over 80% of treated patients, however, approximately half still fail to achieve functional independence at 3 months, a pattern referred to as futile recanalization (4, 5). Understanding the biological determinants of this outcome gap is an unmet need in post-EVT care.

Growing evidence suggests that systemic biological perturbations in the acute reperfusion period contribute substantially to post-EVT outcomes. Three pathophysiological pathways have received particular attention. First, post-procedural hyperglycemia promotes blood–brain barrier disruption, oxidative stress, and thromboinflammation, and has been independently associated with worsened functional outcome and increased hemorrhagic complications after EVT (6, 7). Second, elevated neutrophil-to-lymphocyte ratio (NLR), a marker of systemic inflammatory imbalance, predicts unfavorable outcome and symptomatic intracranial hemorrhage (sICH) following successful recanalization, even after adjustment for stroke severity and reperfusion grade (8–11). Third, D-dimer, a fibrin degradation product reflecting coagulation-fibrinolysis activation, has been associated with large vessel occlusion, cardioembolic stroke etiology, and adverse post-EVT outcomes including sICH and futile recanalization (12–15).

These biomarkers have consistently been studied as independent predictors, analyzed separately through conventional regression approaches. This single-biomarker paradigm has a fundamental limitation: inflammatory activation, metabolic derangement, and hypercoagulability may not occur in isolation but rather co-exist in distinct constellations that differ meaningfully between patients. Patients sharing similar neurological severity at presentation may differ substantially in their underlying biological profile, and such heterogeneity could explain why outcomes vary even among those achieving successful recanalization. A framework capable of capturing co-occurring biological patterns across these pathways may therefore provide prognostic information beyond what individual biomarkers or standard clinical variables can offer.

Latent class analysis (LCA) is a model-based clustering approach that identifies underlying patient subgroups from observed patterns across multiple variables simultaneously (16). It has previously been applied to identify biologically coherent subgroups in critical care syndromes such as sepsis and acute respiratory distress syndrome, with findings that have informed the understanding of treatment response heterogeneity (17, 18). To date, no study has applied LCA to simultaneously integrate inflammatory, metabolic, and coagulatory biomarkers with clinical characteristics to identify biologically distinct phenotypes among EVT-treated patients.

We therefore conducted a retrospective cohort study applying LCA to a consecutive series of EVT-treated patients, using routine post-procedural laboratory and clinical indicators. The primary objective was to identify biologically coherent phenotypes and evaluate their association with 3-month functional outcome. An independent temporal validation cohort was used to assess the reproducibility of the derived phenotypes.

## Materials and methods

### Study design and patient population

This was a retrospective observational cohort study conducted at Taizhou Hospital of Zhejiang Province (affiliated with Wenzhou Medical University), a tertiary care center serving the Taizhou region of Zhejiang Province, China. We retrospectively analyzed data from consecutive patients with acute ischemic stroke who underwent endovascular thrombectomy (EVT) between January 2021 and December 2024. All clinical data were extracted from a standardized stroke database, including baseline demographics, medical history, laboratory parameters, imaging findings, treatment variables, and functional outcomes; this registry has been described previously in the context of a related analysis of nasogastric tube timing and stroke-associated pneumonia (19). The study protocol was approved by the Institutional Review Board of Taizhou Hospital of Zhejiang Province (approval number: K20260121), and the requirement for informed consent was waived due to the retrospective nature of the study.

Patients were eligible if they: (1) presented with acute ischemic stroke confirmed by neuroimaging; (2) underwent EVT with stent retriever, aspiration, or a combination technique; (3) had baseline clinical and laboratory data available within 24 h after the procedure; and (4) had 3-month functional outcome assessed by the modified Rankin Scale (mRS). Patients were excluded if they: (1) had unsuccessful vascular recanalization (mTICI < 2b); (20) (2) lacked 3-month follow-up data; (3) had undergone multiple EVT procedures within 1 year; or (4) had insufficient laboratory values for LCA (> 2 of the 6 continuous indicators missing and not recoverable by imputation). A total of 918 patients were screened, of whom 93 were excluded, yielding a final development cohort of 825 patients (Figure 1). No prior power calculation was performed, consistent with the retrospective, hypothesis-generating nature of this analysis; the analytic sample comprised all consecutive patients meeting the inclusion criteria during the study period. This sample size (*n* = 825) exceeds the threshold of 500 associated with stable latent class enumeration in methodological simulation studies (16).

**Figure 1:**
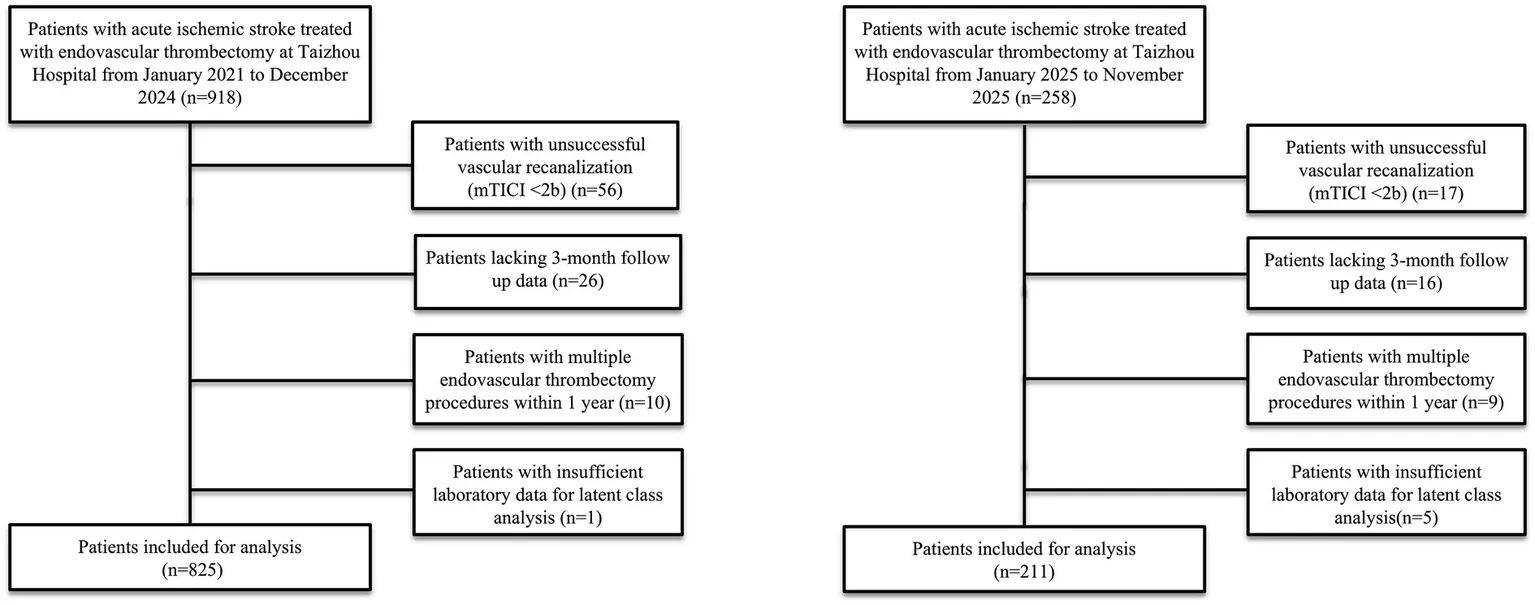
Study flow diagram. Consecutive patients with acute ischemic stroke who underwent endovascular thrombectomy were screened for eligibility at Taizhou Hospital of Zhejiang Province. Exclusion criteria were applied sequentially. The left panel shows derivation of the development cohort (January 2021 to December 2024); the right panel shows derivation of the independent temporal validation cohort (January 2025 to November 2025). Numbers in parentheses indicate patients excluded at each step. *EVT*, endovascular thrombectomy; *mTICI*, modified Thrombolysis in Cerebral Infarction.

### External validation cohort

To assess the generalizability of the derived LCA phenotypes, an independent temporal validation cohort was assembled from the same institution comprising consecutive EVT patients enrolled between January 2025 and November 2025, using identical inclusion and exclusion criteria. A total of 258 patients were screened, of whom 47 were excluded, yielding a validation cohort of 211 patients (Figure 1). This cohort was collected after the development cohort and was not used in any aspect of model derivation or class labeling. As with the development cohort, no prior power calculation was performed for the validation cohort, consistent with the retrospective, hypothesis-generating nature of this analysis. This validation step was designed to test whether the phenotype structure and its association with functional outcome were reproduced in a temporally independent sample, rather than to serve as a separately powered study.

### Data collection

Demographic characteristics, vascular risk factors, premorbid functional status, neuroimaging parameters, procedural details, and in-hospital clinical events were extracted from a standardized stroke database. Laboratory values collected within 24 h after the procedure included white blood cell count (WBC), neutrophil and lymphocyte counts (for calculation of the neutrophil-to-lymphocyte ratio [NLR]), blood glucose, D-dimer, albumin, fibrinogen, platelet count, hemoglobin, creatinine, and lipid panel. Stroke etiology was classified according to the Trial of ORG 10172 in Acute Stroke Treatment (TOAST) criteria (21). Reperfusion grade was assessed using the modified Thrombolysis in Cerebral Infarction (mTICI) scale; successful recanalization was defined as mTICI grade 2b or greater (20), and patients who failed to achieve this were excluded from the analysis.

### Primary outcome

The primary outcome was favorable functional outcome at 3 months, defined as an mRS score of 0–2 (22). Three-month mRS was assessed by trained neurologists or research nurses during outpatient follow-up or by structured telephone interview, blinded to the LCA classification.

### Latent class analysis

LCA was performed to identify underlying patient phenotypes based on a pre-specified set of 12 clinical and laboratory indicators. Variable selection was guided by: (1) pathophysiological relevance to inflammatory and coagulatory mechanisms post-EVT; (2) availability in routine clinical practice; and (3) avoidance of multicollinearity (Spearman |r| < 0.7 between all pairs, verified prior to modeling; Supplementary Figure S1). The final indicator set comprised six binary variables (sex, hypertension, atrial fibrillation, prior stroke, IV thrombolysis, and anterior circulation territory) and six continuous variables (age, admission National Institutes of Health Stroke Scale [NIHSS] score, NLR, admission blood glucose, D-dimer, and albumin).

Missing laboratory values were imputed using median imputation within the development cohort prior to LCA. Models with K = 2 to K = 4 latent classes were estimated using the depmixS4 package (23) (version 1.5.1) in R (version 4.5.0; R Foundation for Statistical Computing, Vienna, Austria), which supports finite mixture models with mixed indicator types. Binary indicators were modeled using multinomial distributions, and continuous indicators were modeled using Gaussian distributions; continuous variables were entered on their original measurement scales, as the Gaussian emission model estimates class-specific means and variances for each indicator independently, thereby accommodating different measurement units without requiring prior standardization (23). Each model was initialized with 50 random starting values to avoid local maxima, retaining the solution with the highest log-likelihood, consistent with methodological guidance recommending at least 50 to 100 independent starting values, particularly for models with more classes (24). Solution stability was further assessed by independently re-fitting each model under four independent random seeds (Supplementary Table S1). Likelihood ratio tests (VLMR) were not computed, as their validity for mixture models with mixed continuous and binary indicator distributions remains uncertain (16, 25).

Model selection was based on the Bayesian Information Criterion (BIC), Akaike Information Criterion (AIC), sample-size adjusted BIC (SABIC), entropy (a measure of classification certainty, with values ≥ 0.70 considered adequate), and the minimum average posterior probability (MinAvePP) of class assignment (Table 1) (16, 26). The K = 3 solution was selected based on a substantial improvement in BIC over K = 2 and acceptable entropy (0.745) and MinAvePP (0.883). Latent classes were subsequently relabeled in descending order of observed favorable outcome rate to facilitate clinical interpretation, with Class 1 representing the most favorable phenotype. Class labels were assigned based on the clinical profile of each latent class and are described in the Results (Table 2).

**Table 1:** Latent class analysis model fit statistics for K = 2 to 4 classes.

Fit statistic	K = 2	K = 3	K = 4
AIC	34,162.5	33,723.9	33,479.8
BIC	34,336.9	33,988.0	33,833.5
SABIC	34,219.4	33,810.1	33,595.3
Entropy	0.813	0.745	0.741
Min. AvePP	0.943	0.883	0.821
Class sizes, n (%)	613 (74.3%) 212 (25.7%)	366 (44.4%) 352 (42.7%) 107 (13.0%)	123 (14.9%) 364 (44.1%) 240 (29.1%) 98 (11.9%)

AIC, Akaike information criterion; BIC, Bayesian information criterion; SABIC, sample-size-adjusted BIC; Entropy, overall classification certainty (range 0–1, higher = better); Min. AvePP, minimum average posterior probability across classes (≥0.70 acceptable, ≥0.80 good); K = 3 was selected based on the largest BIC improvement over K = 2 (ΔBIC = 349) with acceptable entropy and class interpretability.

**Table 2:** Baseline characteristics of patients stratified by LCA phenotype class.

Variable	Overall (*n* = 825)	Class 1 (*n* = 366)	Class 2 (*n* = 352)	Class 3 (*n* = 107)	*p* value
Demographics					
Age, median [IQR], years	70 [62–78]	69 [60–76]	72 [61–79]	73 [67–81]	<0.001
Male sex, n (%)	492 (59.6)	239 (65.3)	191 (54.3)	62 (57.9)	0.010
Vascular risk factors					
Hypertension, n (%)	488 (59.2)	213 (58.2)	216 (61.4)	59 (55.1)	0.458
Diabetes mellitus, n (%)	157 (19.0)	37 (10.1)	101 (28.7)	19 (17.8)	<0.001
Atrial fibrillation, n (%)	328 (39.8)	118 (32.2)	157 (44.6)	53 (49.5)	<0.001
Prior stroke, n (%)	183 (22.2)	70 (19.1)	88 (25.0)	25 (23.4)	0.158
Smoking, n (%)	194 (23.5)	111 (30.3)	72 (20.5)	11 (10.3)	<0.001
Hyperlipidemia, n (%)	38 (4.6)	19 (5.2)	15 (4.3)	4 (3.7)	0.754
Ischemic heart disease, n (%)	50 (6.1)	17 (4.6)	26 (7.4)	7 (6.5)	0.298
Admission characteristics					
NIHSS, median [IQR]	13 [9–18]	12 [8–15]	17 [11–21]	16 [11–21]	<0.001
Systolic BP, median [IQR], mmHg	151 [134–167]	151 [134–165]	151 [134–168]	150 [139–165]	0.734
Diastolic BP, median [IQR], mmHg	86 [76–96]	86 [76–95]	87 [76–98]	81 [75–94]	0.077
Anterior circulation, n (%)	741 (89.8)	346 (94.5)	306 (86.9)	89 (83.2)	<0.001
Stroke etiology, n (%)					<0.001
Large artery atherosclerosis	464 (56.2)	229 (62.6)	182 (51.7)	53 (49.5)	
Cardioembolism	336 (40.7)	119 (32.5)	164 (46.6)	53 (49.5)	
Other/undetermined	25 (3.0)	18 (4.9)	6 (1.7)	1 (0.9)	
Laboratory values					
WBC, median [IQR], ×10^9^/L	8.74 [7.02–10.70]	8.22 [6.60–10.10]	8.95 [7.20–11.30]	9.50 [7.75–11.73]	<0.001
Hemoglobin, median [IQR], g/L	124 [113–135]	126 [116–137]	124 [113–135]	120 [110–128]	0.001
Platelet, median [IQR], ×10^9^/L	191 [154–231]	198 [160–232]	190 [151–231]	179 [147–233]	0.072
NLR, median [IQR]	7.00 [4.40–11.08]	5.48 [3.73–7.89]	8.68 [5.21–13.36]	9.66 [5.75–21.74]	<0.001
Blood glucose, median [IQR], mmol/L	6.90 [5.80–8.25]	6.40 [5.62–7.13]	7.96 [6.30–10.37]	6.80 [6.00–7.95]	<0.001
D-dimer, median [IQR], mg/L	1.20 [0.61–2.21]	0.70 [0.46–1.19]	1.65 [0.82–2.50]	5.83 [4.46–9.57]	<0.001
Albumin, median [IQR], g/L	35.80 [33.70–38.20]	35.90 [34.40–38.18]	35.80 [33.30–38.10]	35.50 [32.90–38.45]	0.083
Fibrinogen, median [IQR], g/L	3.07 [2.61–3.70]	3.06 [2.64–3.63]	3.15 [2.65–3.73]	2.93 [2.39–3.71]	0.095
Creatinine, median [IQR], μmol/L	66 [55–80]	65 [54–77]	66 [55–82]	67 [56–86]	0.272
Total cholesterol, median [IQR], mmol/L	4.01 [3.38–4.67]	4.03 [3.45–4.67]	4.03 [3.34–4.69]	3.80 [3.32–4.56]	0.371
LDL cholesterol, median [IQR], mmol/L	2.18 [1.70–2.69]	2.22 [1.75–2.80]	2.17 [1.67–2.68]	1.90 [1.64–2.50]	0.050
Treatment and procedure					
IV thrombolysis, n (%)	188 (22.8)	57 (15.6)	93 (26.4)	38 (35.5)	<0.001
ORT, median [IQR], min	434 [305–710]	463 [310–785]	410 [298–670]	427 [303–574]	0.074
PRT, median [IQR], min	63 [44–98]	60 [43–100]	65 [45–100]	62 [40–90]	0.228
mTICI 3 reperfusion, n (%)	644 (78.1)	281 (76.8)	275 (78.1)	88 (82.2)	0.485
Outcomes					
Favorable outcome (mRS 0–2), n (%)	460 (55.8)	259 (70.8)	158 (44.9)	43 (40.2)	<0.001
sICH, n (%)	40 (4.8)	6 (1.6)	25 (7.1)	9 (8.4)	0.001
Any ICH, n (%)	131 (15.9)	46 (12.6)	64 (18.2)	21 (19.6)	0.063
Pneumonia, n (%)	290 (35.2)	99 (27.0)	140 (39.8)	51 (47.7)	<0.001
Hospital stay, median [IQR], days	10 [8–13]	10 [8–12]	10 [8–15]	11 [8–16]	0.002
ICU stay, median [IQR], days	1 [1–2]	1 [1–1]	1 [1–3]	1 [1–2]	<0.001

Class 1: Low-burden phenotype; Class 2: Metabolic phenotype; Class 3: Hypercoagulable phenotype. NIHSS, National Institutes of Health Stroke Scale; BP, blood pressure; NLR, neutrophil-to-lymphocyte ratio; WBC, white blood cell; sICH, symptomatic intracranial hemorrhage; ICH, intracranial hemorrhage; ORT, onset-to-reperfusion time; PRT, puncture-to-reperfusion time; mTICI, modified Thrombolysis in Cerebral Infarction.

Each patient in both the development and validation cohorts was assigned to the most probable latent class using posterior probabilities derived from the development cohort model parameters. The same classification algorithm was applied to the validation cohort without re-estimation.

### Statistical analysis

Continuous variables are reported as median [interquartile range, IQR] and compared across classes using the Kruskal-Wallis test. Categorical variables are reported as frequency (percentage) and compared using the chi-squared test or Fisher exact test, as appropriate.

Multivariable logistic regression was used to examine the association between LCA phenotype class and favorable outcome (mRS 0–2) at 3 months, with Class 1 (Low-burden phenotype) as the reference category. Covariates were selected *a priori* based on established predictors of functional outcome after EVT: age, sex, admission NIHSS, IV thrombolysis, mTICI 3 reperfusion, atrial fibrillation, and systolic blood pressure. mTICI 2b or greater was defined as successful recanalization, and mTICI 3 was defined as complete recanalization; because mTICI 3 has been independently associated with outcome (27), it was retained as a covariate. Although age, sex, admission NIHSS, and atrial fibrillation also contributed to LCA derivation, they were retained as covariates given their well-established and independent prognostic roles in EVT outcomes. Biomarker variables that constitute the defining features of each phenotype (NLR, blood glucose, D-dimer, and albumin), as well as comorbidities closely correlated with phenotype membership (diabetes mellitus, hypertension), were excluded from the regression model to avoid circular adjustment. Results are expressed as odds ratios (OR) with 95% confidence intervals (CI).

The area under the receiver operating characteristic curve (AUC) was calculated for a base model containing only established clinical covariates (age, sex, admission NIHSS, IV thrombolysis, mTICI 3, atrial fibrillation, systolic blood pressure), and for the augmented model additionally incorporating LCA phenotype class. AUC comparison was performed using the DeLong method.

In the validation cohort, phenotype class distribution, biomarker profiles, and unadjusted odds ratios for favorable outcome were compared with the development cohort. AUC for LCA class as a sole predictor was calculated to assess discriminative transportability. To assess the stability of the validation cohort estimates given its modest sample size, bootstrap resampling (1,000 replicates, percentile method) was additionally used to compute confidence intervals for the class-outcome odds ratios and for the AUC of LCA class as a discriminator. Heterogeneity of the phenotype-outcome association between the development and validation cohorts was assessed using a pooled logistic regression model with a phenotype × cohort interaction term, tested by likelihood ratio test.

All analyses were performed in R (version 4.5.0). Statistical significance was set at a two-sided *p* < 0.05. This study was reported in accordance with the STROBE guidelines.

## Results

### Patient population

A total of 918 consecutive patients with acute ischemic stroke who underwent EVT were screened between January 2021 and December 2024. After excluding 56 patients with unsuccessful vascular recanalization (mTICI < 2b), 26 lacking 3-month follow-up data, 10 with multiple EVT procedures within 1 year, and 1 with insufficient laboratory data for LCA, 825 patients were included in the development cohort (Figure 1). The median age was 70 years [IQR 62–78], 492 (59.6%) were male, and the median admission NIHSS score was 13 [IQR 9–18]. IV thrombolysis was administered in 188 patients (22.8%), mTICI 3 reperfusion was achieved in 644 (78.1%), and 460 patients (55.8%) achieved a favorable outcome (mRS 0–2) at 3 months. For the validation cohort, 258 patients were screened between January 2025 and November 2025; after excluding 17 with unsuccessful recanalization, 16 lacking follow-up, 9 with multiple procedures, and 5 with insufficient laboratory data, 211 patients were included (Figure 1).

### LCA variable selection

Pairwise Spearman correlation coefficients among the 12 candidate LCA indicators were all below the pre-specified threshold of |r| = 0.70, confirming absence of redundant collinearity (Supplementary Figure S1). The highest observed correlation was between atrial fibrillation and IV thrombolysis (*r* = 0.40). All 12 variables were therefore retained for latent class modeling.

### Latent class model selection

LCA models with K = 2 to K = 4 latent classes were estimated and compared (Table 1). The K = 3 model was selected based on a substantial improvement in BIC over K = 2 (ΔBIC = 349), acceptable entropy (0.745), and adequate minimum average posterior probability (MinAvePP = 0.883), indicating reliable class separation. Although K = 4 further reduced BIC, the improvement was modest (ΔBIC = 154) and the MinAvePP declined to 0.821, approaching the lower acceptable threshold. Across four independently seeded re-fitting attempts, the K = 2 and K = 3 solutions converged identically in every attempt. The K = 4 solution did not converge consistently: one attempt reproduced the partition originally reported in Table 1, while the remaining three attempts converged to a different, mutually similar partition, all with statistically indistinguishable fit (BIC range 33833.48–33833.5; Supplementary Table S1). The K = 3 solution was therefore adopted for all subsequent analyses. Missingness in the four continuous indicators subject to imputation was low (NLR 0%, blood glucose 0.6%, D-dimer 2.3%, albumin 4.7%). A sensitivity analysis comparing the median-imputation-based classification with classifications derived under multiple imputation and random forest imputation showed almost perfect agreement (Cohen’s kappa 0.947 and 0.970, respectively; Supplementary Table S2), indicating that the derived phenotypes are robust to the choice of imputation method.

### Latent class phenotype profiles

The three identified phenotypes are shown in Figure 2 (profile plot) and Table 2 (baseline characteristics). Profile plots showed non-parallel class trajectories across indicators, arguing against a single-spectrum severity gradient.

**Figure 2:**
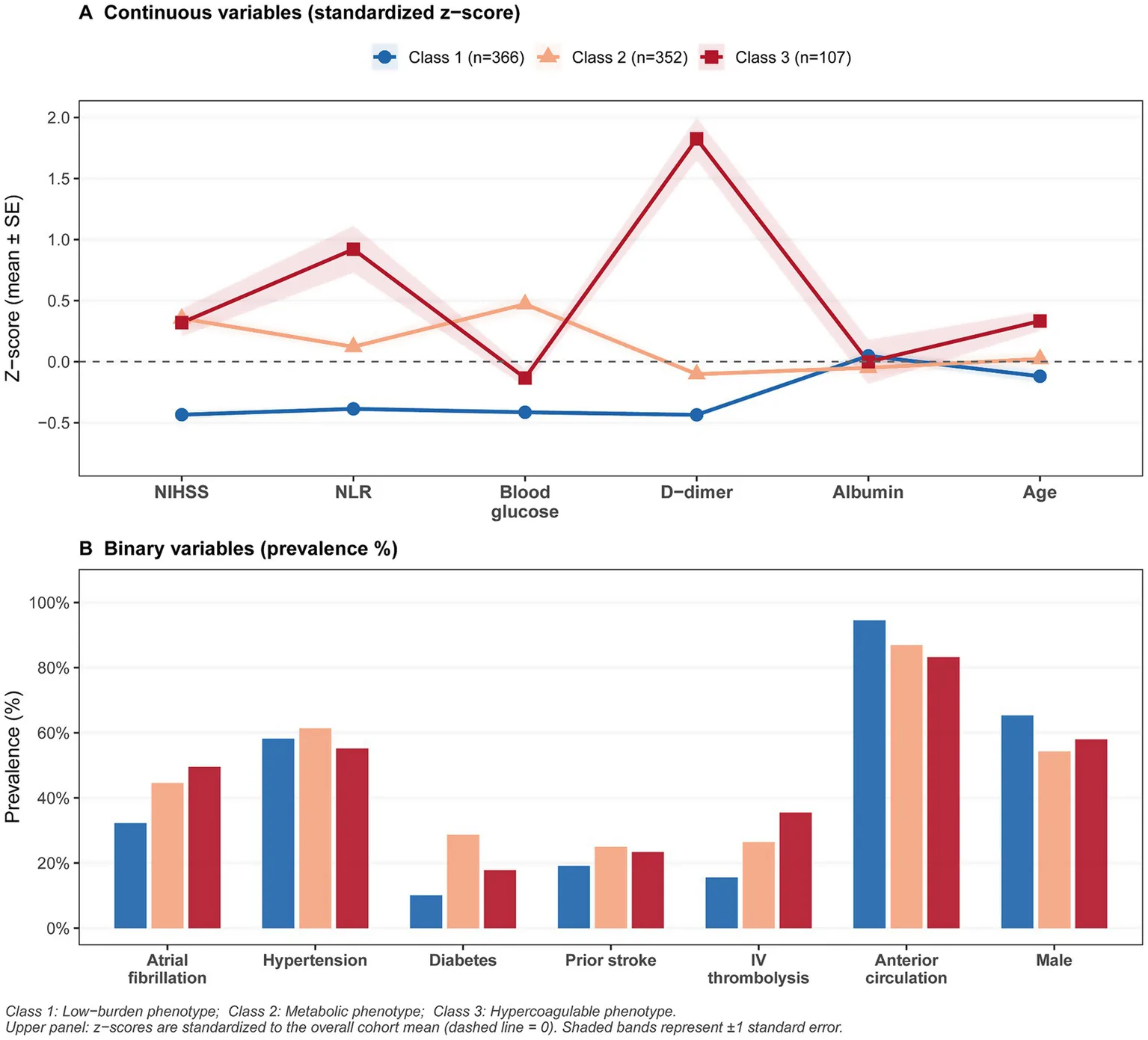
Latent class phenotype profiles. Panel **A** shows standardized z-score profiles of six continuous indicators. Values represent class-specific means ± standard error, standardized to the overall cohort mean (dashed line = 0); shaded bands represent ± 1 standard error. Panel **B** shows the prevalence of the six binary LCA indicators and diabetes mellitus across phenotypes. Class 1: Low-burden phenotype; Class 2: Metabolic phenotype; Class 3: Hypercoagulable phenotype. *NLR*, neutrophil-to-lymphocyte ratio; *IV thrombolysis*, intravenous thrombolysis.

Class 1—Low-burden phenotype (*n* = 366, 44.4%). This class was characterized by the lowest inflammatory and coagulatory burden across all six continuous indicators. Median NLR was 5.48 [IQR 3.73–7.89], blood glucose 6.40 mmol/L [5.62–7.13], D-dimer 0.70 mg/L [0.46–1.19], and albumin 35.9 g/L [34.4–38.2]. This class had the lowest prevalence of atrial fibrillation (32.2%) and diabetes mellitus (10.1%), the highest proportion of anterior circulation strokes (94.5%), and the mildest neurological deficit at admission (median NIHSS 12 (8–15)).

Class 2—Metabolic phenotype (*n* = 352, 42.7%). This class was distinguished by markedly elevated blood glucose (median 7.96 mmol/L [6.30–10.37]) and the highest prevalence of diabetes mellitus (28.7%) among the three classes. NLR was also elevated (median 8.68 [5.21–13.36]). D-dimer was moderately elevated (median 1.65 mg/L [0.82–2.50]), consistent with the higher rates of IV thrombolysis (26.4%) and atrial fibrillation (44.6%) in this group. Admission NIHSS was substantially higher than Class 1 (median 17 (11–21)).

Class 3—Hypercoagulable phenotype (*n* = 107, 13.0%). This class was defined by markedly elevated D-dimer (median 5.83 mg/L [4.46–9.57]), nearly 8-fold higher than Class 1, in the context of the highest NLR (median 9.66 [5.75–21.74]) and oldest age (median 73 years [67–81]). The prevalence of atrial fibrillation was highest in this class (49.5%), and the proportion of anterior circulation strokes was lowest (83.2%). IV thrombolysis was received by 35.5%, the highest of any class. A sensitivity analysis stratifying D-dimer by IV thrombolysis status showed no significant difference within Class 3 (5.58 mg/L among untreated vs. 6.80 mg/L among treated patients, *p* = 0.335), in contrast to Class 1 and Class 2 (both *p* < 0.001), indicating that the D-dimer elevation defining this phenotype was not attributable to thrombolysis-induced fibrinolysis (Supplementary Table S3). Admission NIHSS was 16 (11–21).

### Association of LCA phenotype with functional outcome

Favorable outcome (mRS 0–2) at 3 months differed markedly across phenotypes (Figure 3): 70.8% in Class 1, 44.9% in Class 2, and 40.2% in Class 3 (*p* < 0.001). Rates of symptomatic intracranial hemorrhage (sICH) were 1.6, 7.1, and 8.4% in Classes 1, 2, and 3, respectively (*p* = 0.001). Stroke-associated pneumonia occurred in 27.0, 39.8, and 47.7% of patients across the three classes (*p* < 0.001). On unadjusted logistic regression, both Class 2 (OR 0.34, 95% CI 0.25–0.46, *p* < 0.001) and Class 3 (OR 0.28, 95% CI 0.18–0.43, *p* < 0.001) were strongly associated with unfavorable outcome compared with Class 1.

**Figure 3:**
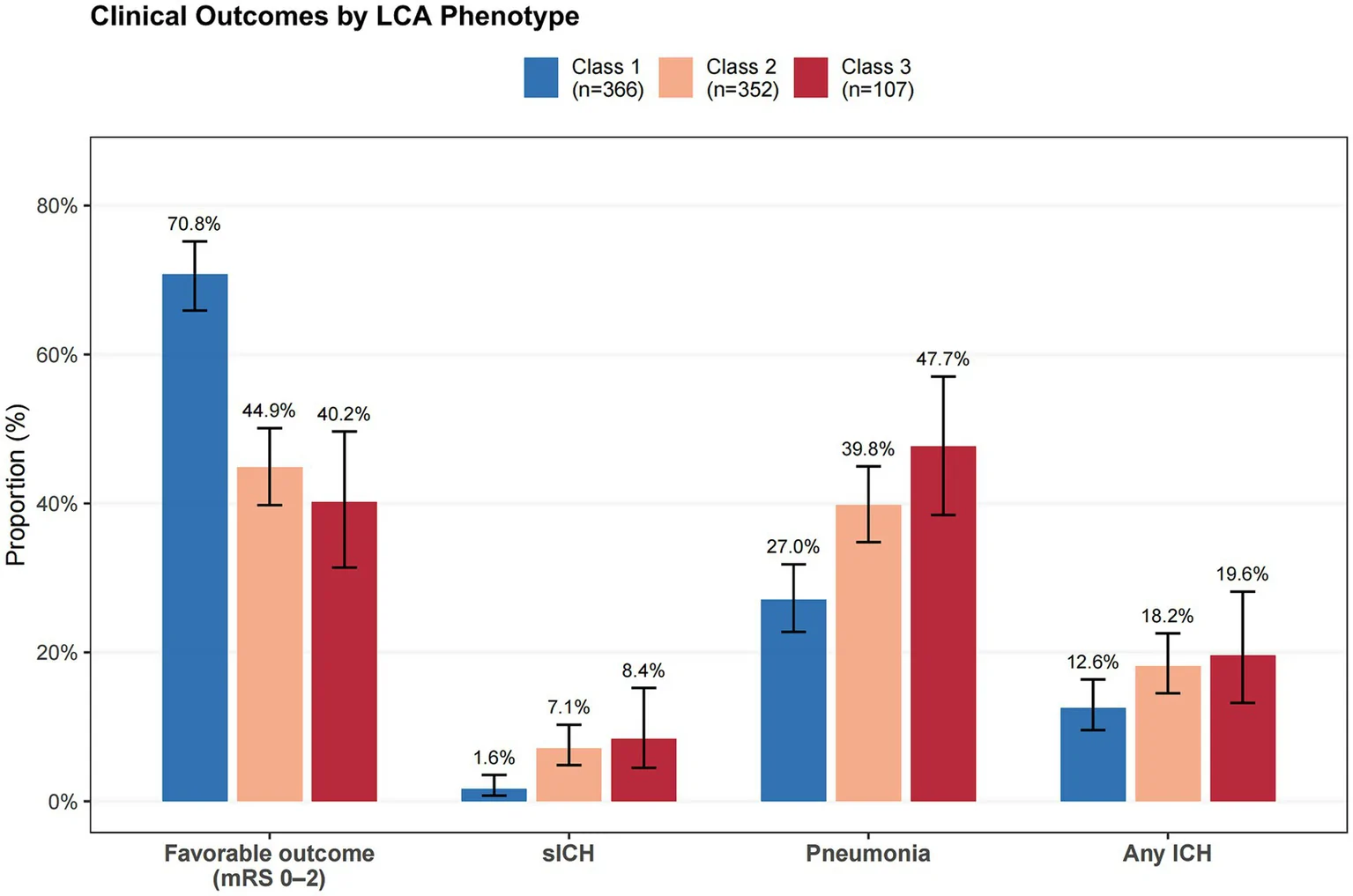
Clinical outcomes by LCA phenotype. Proportions of patients with each outcome are shown by phenotype class, with 95% Wilson confidence intervals. Outcomes were compared across classes using the chi-squared test. *LCA*, latent class analysis; *mRS*, modified Rankin Scale; *sICH*, symptomatic intracranial hemorrhage; *ICH*, intracranial hemorrhage.

After multivariable adjustment for age, sex, admission NIHSS, IV thrombolysis, mTICI 3 reperfusion, atrial fibrillation, and systolic blood pressure, both associations remained statistically significant and clinically meaningful: Class 2 vs. Class 1 OR 0.49 (95% CI 0.35–0.69, *p* < 0.001); Class 3 vs. Class 1 OR 0.41 (95% CI 0.25–0.67, *p* < 0.001; Table 3). A sensitivity analysis removing the four covariates (age, sex, admission NIHSS, atrial fibrillation) that were also used to derive the LCA phenotypes yielded materially similar, and numerically stronger, associations (Class 2 vs. Class 1 OR 0.32, 95% CI 0.23–0.44; Class 3 vs. Class 1 OR 0.25, 95% CI 0.16–0.39; Supplementary Table S4), indicating that adjustment for these covariates did not artificially inflate the phenotype-outcome association. Among the covariates, admission NIHSS (OR 0.92 per point, 95% CI 0.90–0.94) and mTICI 3 reperfusion (OR 1.47, 95% CI 1.02–2.12, *p* = 0.039) were independently associated with outcome, consistent with their established prognostic roles.

**Table 3:** Multivariable logistic regression for favorable outcome (mRS 0–2) at 3 months.

Variable	OR	95% CI	*p* value
LCA phenotype class			
Class 2 vs Class 1	0.49	0.35–0.69	<0.001
Class 3 vs Class 1	0.41	0.25–0.67	<0.001
Covariates			
Age (per year)	0.97	0.96–0.98	<0.001
Male sex	0.91	0.66–1.24	0.546
Admission NIHSS (per point)	0.92	0.90–0.94	<0.001
IV thrombolysis	1.43	0.99–2.08	0.055
mTICI 3 reperfusion	1.47	1.02–2.12	0.039
Atrial fibrillation	1.04	0.74–1.46	0.821
Systolic BP (per mmHg)	1.00	0.99–1.00	0.418

Reference category: class 1 (low-burden phenotype). Model adjusted for age, sex, admission NIHSS, IV thrombolysis, mTICI 3 reperfusion, atrial fibrillation, and systolic blood pressure. OR, odds ratio; CI, confidence interval; NIHSS, National Institutes of Health stroke scale; mTICI, modified thrombolysis in cerebral infarction; BP, blood pressure.

### Discriminative performance

The base model incorporating seven established clinical predictors (age, sex, admission NIHSS, IV thrombolysis, mTICI 3 reperfusion, atrial fibrillation, and systolic blood pressure) yielded an AUC of 0.727 (95% CI 0.692–0.761). Addition of LCA phenotype class to this model improved discrimination to an AUC of 0.743 (95% CI 0.710–0.776; DeLong test *p* = 0.041), representing an absolute improvement of 0.016 (Supplementary Figure S2).

### External validation

The temporal validation cohort comprised 211 EVT patients. Class distribution was closely comparable to the development cohort: Class 1 (Low-burden) 47.4% vs. 44.4%, Class 2 (Metabolic) 42.2% vs. 42.7%, and Class 3 (Hypercoagulable) 10.4% vs. 13.0% (Supplementary Table S5). Biomarker profiles within each class remained consistent across cohorts, with median D-dimer in Class 3 measuring 8.1 mg/L [5.5–11.4] in the validation cohort versus 5.8 mg/L [4.5–9.6] in the development cohort, and median blood glucose in Class 2 measuring 7.9 mmol/L [6.6–9.7] versus 8.0 mmol/L [6.3–10.4], respectively.

The direction and rank-order of outcome associations were consistent between the development and validation cohorts, although the magnitude of favorable outcome rates differed (Class 2: 44.9% vs. 29.2%; Class 3: 40.2% vs. 22.7%), consistent with the modest sample size and wide confidence intervals in the validation cohort. A formal test for heterogeneity (phenotype × cohort interaction) was not statistically significant (*p* = 0.130), providing no strong evidence that the underlying association differs between cohorts. Favorable outcome rates were 70.0% (Class 1), 29.2% (Class 2), and 22.7% (Class 3). On unadjusted logistic regression, Class 2 and Class 3 remained significantly associated with unfavorable outcome compared with Class 1 (Class 2: OR 0.18, 95% CI 0.09–0.33, *p* < 0.001; Class 3: OR 0.13, 95% CI 0.04–0.35, *p* < 0.001). In the development cohort, LCA phenotype class as a sole predictor yielded an AUC of 0.640 (95% CI 0.606–0.675), which improved to 0.716 (95% CI 0.653–0.779) in the validation cohort, with overlapping confidence intervals indicating no attenuation of discriminative performance (Supplementary Table S5). Bootstrap resampling yielded 95% confidence intervals closely consistent with the asymptotic estimates for both the odds ratios (Class 2 vs. Class 1: 0.09–0.32; Class 3 vs. Class 1: 0.03–0.33) and the AUC (0.657–0.777), supporting the stability of these findings despite the modest validation cohort sample size (Supplementary Table S6).

## Discussion

This study identified three biologically distinct phenotypes among EVT-treated patients using routine post-procedural laboratory and clinical indicators. The phenotypes differed markedly in inflammatory burden, metabolic profile, and coagulatory activation, and were independently associated with 3-month functional outcome in both the development and validation cohorts.

The hallmark of Class 2 was post-procedural hyperglycemia and a high prevalence of diabetes mellitus. Elevated blood glucose after EVT promotes blood–brain barrier disruption, augments oxidative stress, and sustains thromboinflammation during the reperfusion phase, thereby increasing the severity of hemorrhagic transformation (6). In the large MR CLEAN Registry, hyperglycemia on admission was independently associated with a shift toward worse 90-day functional outcome and nearly double the risk of symptomatic intracranial hemorrhage after endovascular treatment, irrespective of reperfusion status (7). Consistent with these data, sICH rates in our Class 2 patients were 7.1%, more than four times the rate in Class 1 (1.6%). The co-occurrence of elevated NLR in Class 2 suggests a combined metabolic-inflammatory profile in which systemic inflammation amplifies glucose-mediated neurovascular injury, a synergy that single-biomarker analyses may not capture (28).

Class 3 was distinguished by D-dimer levels nearly eight-fold higher than Class 1, combined with the highest NLR, oldest age, and greatest atrial fibrillation prevalence. Elevated D-dimer reflects ongoing activation of coagulation and fibrinolysis, and is a recognized surrogate of the prothrombotic state associated with atrial fibrillation, correlating with atrial thrombus formation, cerebral infarction volume, and thromboembolic risk (12, 29). In EVT-treated patients, elevated admission D-dimer has been independently associated with increased sICH risk, (13) and post-procedural D-dimer levels have been associated with poor functional outcomes and mortality (14), as well as with futile recanalization (15). These findings may reflect persistent microvascular thrombotic activity that could contribute to limited functional recovery despite successful macrovascular recanalization. In our cohort, favorable outcome was achieved in only 40.2% of Class 3 patients in the development cohort and 22.7% in the validation cohort, the lowest of any class, despite the highest rates of IV thrombolysis use (35.5%). This raises the possibility that the underlying hypercoagulable milieu may limit the benefit of reperfusion therapies in this subgroup, though prospective data are needed to test this hypothesis.

Elevated NLR was present in both Class 2 and Class 3, consistent with evidence that post-EVT neuroinflammation contributes to futile reperfusion (8). After recanalization, neutrophil activation generates reactive oxygen species and neutrophil extracellular traps that disrupt the blood–brain barrier, exacerbate cerebral edema, and promote hemorrhagic transformation (9). NLR measured within 24 h of the procedure has been shown to independently predict unfavorable outcome and sICH after accounting for reperfusion status, stroke severity, and age, (8, 10) and to predict intracranial hemorrhage after EVT independent of established clinical risk factors (11). In the present cohort, elevated NLR co-existed with distinct biological contexts: a metabolic-hyperglycemic profile in Class 2 and a prothrombotic profile in Class 3. This pattern suggests that the inflammatory signal may carry different prognostic implications depending on the broader biological milieu. Whether these different constellations require targeted therapeutic strategies warrants prospective investigation.

The distinction between Class 2 and Class 3 warrants further comment. While NLR was elevated in both classes and D-dimer and age increased in a broadly graded fashion from Class 1 to Class 3, blood glucose did not follow this pattern: median blood glucose rose from 6.40 mmol/L in Class 1 to 7.96 mmol/L in Class 2 but fell to 6.80 mmol/L in Class 3, close to the Class 1 level. This non-monotonic profile argues against interpreting the three classes as points along a single linear severity continuum, and instead supports the presence of two partially distinct biological processes underlying Class 2 and Class 3, a metabolic-inflammatory constellation and a coagulation-inflammatory constellation respectively, that share elevated inflammatory burden but diverge in their metabolic and coagulation features.

The class distribution, biomarker profiles, and direction of outcome associations were closely replicated in the 2025 temporal validation cohort, supporting the reproducibility of the derived phenotypes within this institution. Class proportions remained stable within 3 percentage points across cohorts, and the rank-order of favorable outcome rates was preserved. Addition of LCA class to a conventional clinical model improved AUC from 0.727 to 0.743 (DeLong test *p* = 0.041), a statistically significant but modest increment (ΔAUC = 0.016). This finding suggests that phenotype-related biological differences contribute incrementally to outcome prediction, though the small magnitude of improvement indicates that LCA class should be interpreted alongside, rather than independent of, established clinical predictors.

These findings are exploratory and hypothesis-generating. Phenotype assignment using routine post-procedural laboratory values may offer a framework for early risk stratification, potentially motivating future prospective studies of intensified monitoring in Classes 2 and 3, rather than supporting any immediate change in clinical practice. For Class 2, in which hyperglycemia co-occurs with elevated NLR, glycemic control during the peri-reperfusion period represents a testable intervention target, building on prior evidence linking admission hyperglycemia to blood–brain barrier disruption and hemorrhagic transformation after EVT. For Class 3, the markedly elevated D-dimer and high atrial fibrillation prevalence raise the question of whether the timing, agent, or intensity of anticoagulation after EVT could modify the hypercoagulable state and its association with poor outcome; this would require prospective study given the competing risk of hemorrhagic transformation in the acute post-EVT period. Neither hypothesis can be tested with the present retrospective design. Before either could inform the design of an intervention study, the phenotype-outcome associations reported here would need prospective validation. At this stage, these observations are hypothesis-generating and do not constitute evidence that such interventions are already warranted. The modest AUC increment cautions against overstating the incremental predictive value of LCA class over established clinical predictors.

This study has several limitations. First, the single-center retrospective design limits generalizability, and prospective multicenter validation is needed. Second, the biomarkers were measured within 24 h after EVT, and it is unclear to what extent the observed profiles reflect pre-existing patient characteristics versus peri-procedural or early post-reperfusion biology. In addition, the exact sampling time within this 24-h window was not standardized or recorded; evidence from other cohorts suggests that the predictive value of inflammatory markers such as NLR may vary across this interval, (30) and the observed biomarker levels may have been influenced by reperfusion injury or evolving complications. The biological interpretation of the identified phenotypes should accordingly be considered provisional. Third, although a temporal validation cohort was used, it was drawn from the same institution, which does not constitute true external validation. In the validation cohort, only 22 patients were classified as the Hypercoagulable phenotype (Class 3), which limits the precision of subgroup-specific estimates in this class and underscores the need for external validation in a larger, ideally multicenter, sample. Fourth, residual confounding cannot be excluded in a non-randomized design. Fifth, NLR and D-dimer can be elevated by conditions other than the stroke and reperfusion process itself, including infection, malignancy, recent surgery, and venous thromboembolism; these conditions were not systematically captured in our database and could not be adjusted for, which may have introduced additional, non-differential variability into the biomarker-based phenotypes. Sixth, baseline antiplatelet and anticoagulant medication use was not systematically captured in our database and could not be examined as a potential confounder, particularly relevant to the Hypercoagulable phenotype (Class 3, 49.5% atrial fibrillation), in which preprocedural anticoagulation status may influence both D-dimer levels and sICH risk. Seventh, no *a priori* power calculation was performed for the primary analysis of LCA phenotype and functional outcome; while the development cohort (*n* = 825) exceeds the sample size of 500 associated with stable class enumeration in general LCA simulation studies (16), this threshold is derived from external simulation literature rather than data-specific validation, and our retrospective, single-center design did not allow for independent confirmation of enumeration stability at a larger sample size. The study was therefore not formally powered to detect between-class differences in the primary outcome, and underpowered subgroup comparisons, particularly for the smallest phenotype (Class 3), cannot be excluded. Finally, the entropy of 0.745 and MinAvePP of 0.883, while meeting accepted thresholds, indicate that classification uncertainty exists for some patients, and the clinical implications of misclassification in boundary cases are unknown.

## Conclusion

LCA identified three clinically and biologically distinct phenotypes among EVT-treated patients: a Low-burden phenotype with favorable outcomes, a metabolic phenotype characterized by hyperglycemia and combined inflammatory activation, and a Hypercoagulable phenotype defined by markedly elevated D-dimer and atrial fibrillation. Both the Metabolic and Hypercoagulable phenotypes were independently associated with unfavorable 3-month outcome, and these associations were reproduced in an independent temporal validation cohort. These findings demonstrate that EVT-treated patients encompass biologically distinct subgroups, and that routine post-procedural laboratory values may carry prognostic information not fully captured by established clinical predictors. Multicenter prospective validation is needed before these phenotypes can inform clinical practice.

## Data Availability

The datasets analyzed during the current study are not publicly available due to ethical and privacy concerns. Data may be shared upon reasonable request to the corresponding author, subject to institutional approval and appropriate data use agreements.
