# Supplementary material for: Latent class analysis identifies three biologically distinct phenotypes among patients undergoing endovascular thrombectomy: a derivation and temporal validation study
Source: Front Neurol. 2026 Oct 15;17:1855033. doi: 10.3389/fneur.2026.1855033 (PMC13716782; doi:10.3389/fneur.2026.1855033)
Supplement: Supplementary file 1 [file Table_1.DOCX]

**Supplementary Table S1. Stability of latent class solutions (K=2 to K=4) across independent re-fitting attempts**

| **K** | **Run** | **BIC** | **Class sizes, n** |
| --- | --- | --- | --- |
| 2 | 1 | 34336.92 | 613 / 202 |
| 2 | 2 | 34336.92 | 613 / 202 |
| 2 | 3 | 34336.92 | 613 / 202 |
| 3 | 1 | 33987.97 | 366 / 352 / 107 |
| 3 | 2 | 33987.97 | 366 / 352 / 107 |
| 3 | 3 | 33987.97 | 366 / 352 / 107 |
| 4 | 1 | 33833.50 | 123 / 364 / 240 / 98 |
| 4 | 2 | 33833.48 | 249 / 239 / 217 / 120 |
| 4 | 3 | 33833.48 | 250 / 239 / 216 / 120 |
| 4 | 4 | 33833.48 | 251 / 238 / 216 / 120 |

*Each K was independently re-fit under four separate random seeds (50 random starting values retained per fit, as in the primary analysis). The K=2 and K=3 solutions converged to an identical partition in every attempt. The K=4 solution did not converge consistently: one attempt converged to the partition reported in Table 1, while three attempts converged to a different, mutually similar partition; all four attempts achieved statistically indistinguishable BIC. BIC, Bayesian information criterion.*

**Supplementary Table S2. Concordance of LCA phenotype classification under alternative approaches to handling missing data (development cohort, n=825)**

| **Imputation approach** | **Missingness handled (of 4 continuous indicators)** | **Agreement with original classification** | **Cohen's kappa** |
| --- | --- | --- | --- |
| Median imputation (original) | NLR 0%, glucose 0.6%, D-dimer 2.3%, albumin 4.7% | Reference | Reference |
| Multiple imputation (PMM, MAR, m=10) | as above | 96.8% (95.3–98.8%) | 0.947 (0.922–0.980) |
| Random forest (iterative, missForest-style) | as above | 98.2% | 0.970 |

*Missingness proportions refer to the four continuous LCA indicators (NLR, blood glucose, D-dimer, albumin) prior to imputation. Agreement and kappa computed against the classification derived under the original median-imputation approach used in the primary analysis; values are mean (range) across the 10 multiply imputed datasets. NLR, neutrophil-to-lymphocyte ratio; PMM, predictive mean matching; MAR, missing at random.*

**Supplementary Table S3. D-dimer levels stratified by intravenous thrombolysis status within each LCA phenotype**

| **Class** | **No thrombolysis, n** | **D-dimer, median [IQR]** | **Thrombolysis, n** | **D-dimer, median [IQR]** | ***p* value*** |
| --- | --- | --- | --- | --- | --- |
| Class 1 | 309 | 0.68 [0.44–1.07] | 57 | 1.13 [0.60–1.48] | <0.001 |
| Class 2 | 259 | 1.42 [0.69–2.30] | 93 | 2.14 [1.41–2.85] | <0.001 |
| Class 3 | 69 | 5.58 [3.25–10.26] | 38 | 6.80 [4.84–8.95] | 0.335 |

**p value compares thrombolysis-treated versus untreated patients within each class (Wilcoxon rank-sum test). D-dimer values are median [interquartile range], mg/L. Among Class 3 patients who did not receive thrombolysis (n=69), median D-dimer remained markedly elevated relative to Class 1 (0.70 mg/L overall, p=1.0×10⁻³¹) and Class 2 (1.64 mg/L overall, p=3.3×10⁻²¹). After excluding all thrombolysis-treated patients from the multivariable regression, the association between phenotype and unfavorable outcome remained significant (Class 2 vs Class 1: OR 0.33, 95% CI 0.24–0.47; Class 3 vs Class 1: OR 0.22, 95% CI 0.12–0.38, versus OR 0.41 in the full cohort). IQR, interquartile range.*

**Supplementary Table S4. Sensitivity analysis of the LCA phenotype-outcome association after removing LCA-derived covariates (development cohort, n=825)**

| **Comparison** | **Main model OR (95% CI)** | **Sensitivity model OR (95% CI)** |
| --- | --- | --- |
| Class 2 vs Class 1 | 0.49 (0.35–0.69) | 0.32 (0.23–0.44) |
| Class 3 vs Class 1 | 0.41 (0.25–0.67) | 0.25 (0.16–0.39) |

*Data are OR (95% CI). Main model adjusted for age, sex, admission NIHSS, IV thrombolysis, mTICI 3 reperfusion, atrial fibrillation, and systolic blood pressure, as reported in manuscript Table 3. Sensitivity model adjusted for IV thrombolysis, mTICI 3 reperfusion, and systolic blood pressure only, removing the four covariates (age, sex, admission NIHSS, atrial fibrillation) that were also used to derive the LCA phenotypes. All estimates p<0.001. OR, odds ratio; CI, confidence interval; NIHSS, National Institutes of Health Stroke Scale; mTICI, modified Thrombolysis in Cerebral Infarction.*

**Supplementary Table S5. Comparison of LCA class characteristics between development and validation cohorts**

|  |  | **Development cohort (n=825)** | **Validation cohort (n=211)** |
| --- | --- | --- | --- |
| **Class distribution** |  |  |  |
|  | Class 1, n (%) | 366 (44.4%) | 100 (47.4%) |
|  | Class 2, n (%) | 352 (42.7%) | 89 (42.2%) |
|  | Class 3, n (%) | 107 (13.0%) | 22 (10.4%) |
| **Favorable outcome (mRS 0–2)** | |  |  |
|  | Class 1 | 70.8% | 70.0% |
|  | Class 2 | 44.9% | 29.2% |
|  | Class 3 | 40.2% | 22.7% |
| **Age, years, median [IQR]** |  |  |  |
|  | Class 1 | 69.0 [60.0–76.0] | 70.0 [59.8–77.0] |
|  | Class 2 | 72.0 [61.0–79.0] | 75.0 [67.0–83.0] |
|  | Class 3 | 73.0 [66.5–80.5] | 78.0 [67.8–84.8] |
| **Admission NIHSS, median [IQR]** | |  |  |
|  | Class 1 | 12.0 [8.0–15.0] | 11.0 [7.0–15.0] |
|  | Class 2 | 17.0 [11.0–21.0] | 17.0 [12.0–23.0] |
|  | Class 3 | 16.0 [11.0–21.0] | 15.0 [15.0–22.8] |
| **NLR, median [IQR]** |  |  |  |
|  | Class 1 | 5.5 [3.7–7.9] | 5.2 [3.8–7.0] |
|  | Class 2 | 8.7 [5.2–13.4] | 8.6 [4.9–13.2] |
|  | Class 3 | 9.7 [5.7–21.7] | 7.3 [4.5–13.9] |
| **Blood glucose, mmol/L, median [IQR]** | |  |  |
|  | Class 1 | 6.4 [5.6–7.1] | 6.7 [6.0–7.2] |
|  | Class 2 | 8.0 [6.3–10.4] | 7.9 [6.6–9.7] |
|  | Class 3 | 6.8 [6.0–8.0] | 7.3 [7.0–8.8] |
| **D-dimer, mg/L, median [IQR]** | |  |  |
|  | Class 1 | 0.7 [0.5–1.2] | 0.6 [0.3–1.1] |
|  | Class 2 | 1.6 [0.8–2.5] | 1.6 [0.8–2.3] |
|  | Class 3 | 5.8 [4.5–9.6] | 8.1 [5.5–11.4] |
| **Albumin, g/L, median [IQR]** |  |  |  |
|  | Class 1 | 35.9 [34.4–38.2] | 36.4 [34.3–38.3] |
|  | Class 2 | 35.8 [33.3–38.1] | 35.6 [32.6–37.4] |
|  | Class 3 | 35.5 [32.9–38.5] | 36.1 [34.3–38.8] |
| **Logistic regression (unadjusted OR, 95% CI)** | |  |  |
|  | Class 2 vs Class 1 | 0.34 (0.25–0.46), p<0.001 | 0.18 (0.09–0.33), p<0.001 |
|  | Class 3 vs Class 1 | 0.28 (0.18–0.43), p<0.001 | 0.13 (0.04–0.35), p<0.001 |
| **Discriminative performance** |  |  |  |
|  | AUC (95% CI) | 0.640 (0.606–0.675) | 0.716 (0.653–0.779) |

Data are median [IQR], % or OR (95% CI). Logistic regression models are unadjusted (LCA class as sole predictor). AUC, area under the receiver operating characteristic curve; NLR, neutrophil-to-lymphocyte ratio; OR, odds ratio; CI, confidence interval.

**Supplementary Table S6. Bootstrap 95% confidence intervals for validation cohort estimates (1000 resamples)**

| **Estimate** | **Point estimate** | **Asymptotic 95% CI** | **Bootstrap 95% CI** |
| --- | --- | --- | --- |
| Class 2 vs Class 1, OR | 0.18 | 0.09–0.33 (Wald) | 0.09–0.32 |
| Class 3 vs Class 1, OR | 0.13 | 0.04–0.35 (Wald) | 0.03–0.33 |
| AUC (LCA class) | 0.716 | 0.653–0.779 (DeLong) | 0.657–0.777 |

*Validation cohort, n=211. Bootstrap performed by resampling validation cohort patients with replacement (1000 replicates), holding phenotype classification fixed, and recomputing the logistic regression and ROC AUC in each replicate; 95% CI computed via the percentile method. OR, odds ratio; CI, confidence interval; AUC, area under the receiver operating characteristic curve.*

**Supplementary Figure S1.** Spearman correlation matrix of LCA input variables.

Pairwise Spearman correlation coefficients among the 12 candidate indicators are shown. Color intensity reflects the magnitude of correlation; red indicates positive and blue indicates negative correlation. All pairwise coefficients were below the pre-specified collinearity threshold of |r| = 0.70.

*NLR*, neutrophil-to-lymphocyte ratio; *IV thrombolysis*, intravenous thrombolysis.

**Supplementary Figure S2.** Receiver operating characteristic curves for prediction of favorable outcome.

ROC curves are shown for the conventional clinical model (age, sex, admission NIHSS, IV thrombolysis, mTICI 3 reperfusion, atrial fibrillation, and systolic blood pressure) and for the augmented model additionally incorporating LCA phenotype class. AUC comparison was performed using the DeLong method.

*AUC*, area under the receiver operating characteristic curve; *CI*, confidence interval; *LCA*, latent class analysis; *NIHSS*, National Institutes of Health Stroke Scale; *mTICI*, modified Thrombolysis in Cerebral Infarction.
